# Silver Nanoparticles as Anticancer Agents: Mechanisms Insight, Current Studies, and Limitations

**DOI:** 10.3390/ph19020241

**Published:** 2026-01-30

**Authors:** Erkan Efe Okur, Emir Akdaşçi, Furkan Eker, Mikhael Bechelany, Sercan Karav

**Affiliations:** 1 Department of Molecular Biology and Genetics, Çanakkale Onsekiz Mart University, Çanakkale 17100, Türkiye; erkanefe.okur@gmail.com (E.E.O.); emirakdasci@stu.comu.edu.tr (E.A.); furkan.eker@stu.comu.edu.tr (F.E.); 2 European Institute for Membranes (IEM), UMR-5635, University of Montpellier, ENSCM, CNRS, Place Eugène Bataillon, CEDEX 5, F-34095 Montpellier, France

**Keywords:** metal nanoparticles, antitumor activity, cytotoxicity, selectivity, anticancer mechanisms, tumor microenvironment

## Abstract

Silver nanoparticles (AgNPs) have been studied extensively in recent years due to their biological activities. In addition to their well-known antibacterial, antioxidant, and anti-inflammatory properties, AgNPs also exhibit anticancer properties. Increasing evidence from in vivo and in vitro studies demonstrates that AgNPs exert significant anticancer effects through multiple mechanisms, including oxidative stress, mitochondrial dysfunction, DNA damage, cell cycle arrest, and apoptosis. In addition to these mechanisms, inhibition of certain pathways is also an important mechanism that enables AgNPs to exhibit anticancer activity. Furthermore, green-synthesized AgNPs often exhibit enhanced biocompatibility and improved selective cytotoxicity against cancer cells. Despite these promising findings, concerns regarding AgNP-associated toxicity, non-specific cellular damage, and long-term safety remain major challenges limiting their clinical translation. Strategies such as size and shape optimization, surface functionalization, and combination therapies have been recommended to enhance anticancer efficacy while minimizing adverse effects on healthy cells. This review brings together recent studies, offers a broad perspective, and aims to present an objective viewpoint to fully explain the anticancer potential and current challenges of AgNPs.

## 1. Introduction

Cancer is one of the most important public health issues worldwide. While breast, lung, prostate, and prostate cancer are among the most commonly diagnosed cancers, lung cancer stands out as the leading cause of death worldwide [1,2]. These data demonstrate that the global cancer burden is rapidly increasing and that effective early diagnosis, treatment, and control strategies are critical [3,4]. In addition, conventional treatment approaches such as chemotherapy, radiotherapy, and surgery are among the fundamental practices in cancer management and have been the backbone of clinical practice for many years. However, conventional cancer therapies often suffer from limitations such as off-target toxicity and the emergence of drug resistance, reducing their overall therapeutic efficacy. Eventually, the need for alternative approaches that offer improved selectivity and safety profiles is increasing day by day.

Nanoparticles (NPs) are generally defined in the 1–100 nm size range and are distinguished from conventional materials by the unique physical and chemical properties they exhibit at this scale [5]. Metal NPs have attracted significant attention in recent years across various disciplines and technological fields due to their unique physicochemical properties. The ability to functionalize their size, shape, and surface characteristics at the nanoscale can greatly influence their optical, electrical, catalytic, and biological behavior. In particular, metal NPs such as silver, gold, copper, zinc, and iron oxide offer significant potential in biomedical applications due to their high surface area/volume ratio [6,7]. Nanotechnological advances may offer alternative treatment ways for the rising cancer-related deaths. In this context, silver nanoparticles (AgNPs) stand out as a promising anticancer agent with their unique physicochemical properties, cytotoxic effects against cancer cells, and selective toxicity depending on the synthesis method [8].

AgNPs are biologically active NPs with sizes generally ranging from 1 to 100 nm [9]. Diverse biologic activities of AgNPs occur thanks to their various physicochemical properties and have a strong potential to be used in many different fields such as biomedical applications, food technologies, drug delivery systems, and dental applications [10,11,12]. Some of the physicochemical properties that affect the biological activity of AgNPs include surface chemistry, size, and shape [13]. Their physicochemical properties, especially their high surface area-to-volume ratios, can provide diverse activities such as antioxidant, antibacterial, anticancer, antifungal, and anti-inflammatory [14]. One of the bases of these biological properties, especially for anticancer activity, of AgNPs is their cytotoxic activity. [15]. This damage to cells can vary depending on the physicochemical properties of the AgNPs. Furthermore, how they distribute in tissues, their penetrating potential, and their ability to be accepted by cells are also important parameters that determine the level of cytotoxicity [16]. It is also known that AgNP can exert dose-dependent toxicity to various types of organisms such as viruses, bacteria, fungi, animal cells, and cancer cells [17]. This wide diversity can generally be attributed to similar mechanisms. AgNPs can be lethal to cells through mechanisms such as inducing reactive oxygen species (ROS) generation, mitochondrial accumulation, and DNA damage [18]. Thus, AgNPs can cause cytotoxicity by activating apoptosis genes in cells, disrupting the cell cycle, causing mitochondrial dysfunction, and increasing ROS production.

Selectivity, that is, the most critical property underlying anticancer activity, is defined as the observation that AgNPs have a much greater toxic effect on cancer cells than on healthy cells [13]. This selectivity arises because the abnormal metabolism of cancer cells enables higher uptake of AgNPs, and cancer cells are also more susceptible to ROS-mediated damage [19]. Additionally, AgNPs’ ability to localize within mitochondria and their functionalizable surface chemistry may contribute to this selectivity [20,21]. In this context, green synthesis methods also contribute significantly to the selectivity of AgNPs. It is widely accepted that AgNPs capped with plant extracts, microbial metabolites, or polyphenols exhibit much more selective toxicity than traditionally synthesized AgNPs [19]. The anticancer properties of AgNPs synthesized via green methods have generally been found to be significantly more active than those of conventionally synthesized AgNPs [22].

AgNPs possess specialized tumor-killing activity as a result of their physicochemical properties, specifically, their small size, diverse shapes, and variable surface chemistry. To prevent this cell-killing effect from harming healthy cells, appropriate synthesis methods and NP modification methods, such as surface functionalization and size and surface chemistry optimization, can enhance the selective toxicity of AgNPs relative to other approaches, thereby demonstrating positive effects on anticancer activity.

This review provides an overview of AgNPs as emerging anticancer agents due to their unique physicochemical properties and cellular mechanisms underlying their cytotoxicity, including overproduction of ROS, DNA damage, mitochondrial dysfunction, and apoptosis induction. Although AgNPs can also be exploited in light-based approaches such as photothermal therapy through their optical properties, leading to cytotoxicity mediated by intracellular temperature increase, therapies involving external light stimulation are beyond the scope of this review. Current in vitro and in vivo studies within this scope are reviewed, including how the toxic effects of AgNPs can be reduced and the concerns and limits associated with this toxicity. By evaluating the advantages and disadvantages of AgNPs, the review aimed to address the role and importance of AgNPs in anticancer treatment studies in a balanced and critical manner.

## 2. Cytotoxicity of AgNPs

Among inorganic NPs, AgNPs have attracted significant attention in biomedical applications in recent years, particularly due to their antimicrobial, antioxidant, and anticancer properties. However, to safely exploit this potential, a detailed understanding of the cytotoxic effects of AgNPs is necessary. Such effects are especially pronounced in cancer cells, where they can trigger apoptosis and necrosis, thereby supporting the applicability of AgNPs as promising anticancer agents [23].

**Figure 1:**
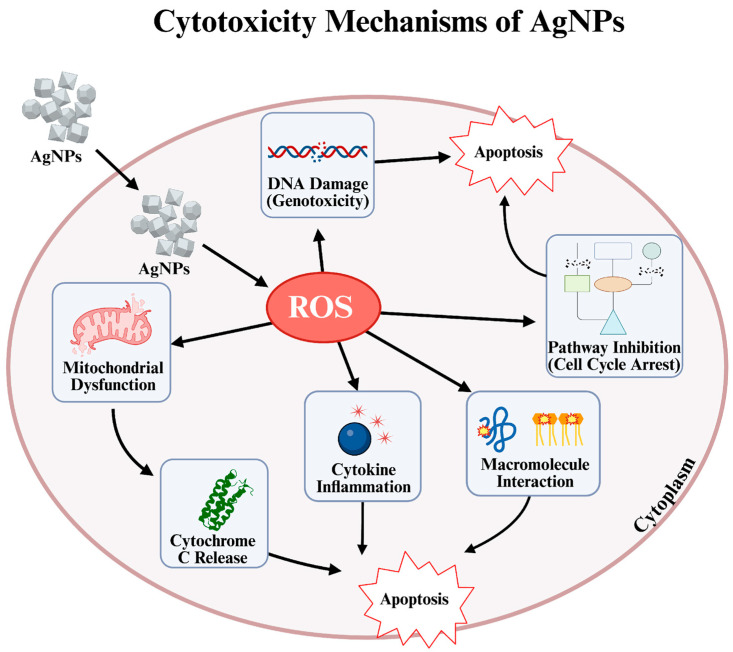
Cytotoxicity mechanisms of AgNPs [29,30,31].

Although the concepts of cytotoxicity and anticancer activity are related, the most fundamental difference between them is target specificity [24]. Cytotoxicity is defined as a substance’s ability to destroy cells regardless of what type of cell they are. In other words, it is a type of “cellular toxicity” that affects both healthy and hazardous cells [25]. Anticancer activity, on the other hand, is a much more refined and targeted form of cytotoxicity effect [26,27]. It defines a substance’s ability to distinguish and prioritize the destruction of cancer cells over healthy cells. In summary, an effective cancer treatment must be cytotoxic against cancer cells. However, what makes a substance valuable is its ability to direct this cytotoxic effect only toward cancerous tissue without causing widespread destruction [28]. Studies have shown that AgNPs exhibit remarkable anticancer potential by inducing cytotoxic effects in various human cell lines. Particularly, small-sized AgNPs demonstrate enhanced ability to trigger apoptosis, necrosis, or autophagy in cancer cells, leading to effective tumor cell elimination (Figure 1). Additionally, their size-dependent interactions can cause DNA strand breaks, ROS-mediated oxidative stress, and modulation of inflammatory cytokines, all contributing to their anticancer efficacy. Therefore, precise control over nanoparticle (NP) dose, size, and exposure conditions is crucial to maximize therapeutic benefits while minimizing toxicity [18].

In a study, various analysis methods, including MTT (3-(4,5-dimethylthiazol-2-yl)-2,5-diphenyltetrazolium bromide) assay, wound healing assay, transwell invasion assay, and DHE (dihydroethidium) staining, were used on AgNPs with sizes of 13 nm, 45 nm, and 92 nm. The results showed that 13 nm AgNPs exhibited the strongest effects on A549 lung cancer cells. Furthermore, they exhibited higher toxicity in A549 cells than in normal cells. Additionally, as AgNPs increased in size, their ability to inhibit cell migration and invasion decreased [32].

Another study observed that spherical AgNPs with a size ranging from 10 to 20 nm had higher anticancer activity on HepG2 liver cancer cells than rod-shaped AgNPs with a size ranging from 50 to 80 nm. This study also emphasized the importance of AgNP morphology, beyond size, in biological activity. Another study by Pucelik et al. examined how both size and surface chemistry affect anticancer activity. Although very high cytotoxicity was observed with NPs smaller than 5 nm, it was noted that selectivity was lost depending on the toxicity that was observed against both cancer and normal cells. A similar effect was observed with 10 nm AgNPs, while 40 nm AgNPs exhibited both the highest anticancer properties and the lowest toxicity against normal cells. Furthermore, AgNPs coated with *N*,*N*,*N*-trimethyl-(11-mercaptoundecyl) ammonium chloride (TMA), a positively charged coating agent, were observed to have lower selectivity but higher cytotoxicity. Larger AgNPs coated with trisodium citrate (TSC), a negative coating agent, were observed to have more selective and stronger anticancer properties [33]. Overall, these findings highlight that the anticancer activity of AgNPs is not a fixed property, but rather a dynamic outcome shaped by their physicochemical characteristics.

In addition to these physicochemical properties, the synthesis method plays a critical role in shaping the cytotoxic potential of AgNPs. For instance, researchers demonstrated that green-synthesized AgNPs from walnut green husk extract exhibited stronger cytotoxicity against MCF-7 breast cancer cells than chemically synthesized AgNPs. The green-synthesized AgNPs also exhibited minimal effects on normal fibroblasts, suggesting enhanced selectivity [19]. Similarly, another research reported that AgNPs generated by electron beam synthesis showed a higher selectivity index for tumor cells when compared to normal fibroblasts [34]. Consistent with these findings, researchers observed that biosynthesized from *C. roseus* aqueous extract AgNPs displayed significant antiproliferative activity against HeLa cells, with comparatively lower toxicity to healthy cells [35]. Studies show that the cytotoxic effects of AgNPs are not exclusive to cancer cells, but can similarly exert strong biological responses in microorganisms. This occurs through multiple mechanisms such as disruption of cell membrane integrity, excessive ROS production, protein and DNA damage, and inhibition of metabolic pathways [29]. Just as in tumor cells, the size, shape, and surface charge of AgNPs are among the key factors determining the intensity and selectivity of this toxic effect in bacterial cells. Therefore, understanding the antibacterial cytotoxicity of AgNPs is crucial both for explaining their mechanisms of action and for preventing off-target effects against bacteria that are beneficial to human health. In this context, a study by Wang et al. investigated the effects of bacterial nanocellulose containing AgNPs on *Lactobacillus rhamnosus*, *Lactobacillus acidophilus*, and *Bifidobacterium animalis*. The findings showed that at high concentrations (≥2000 µg/mL) and in the early stages of incubation, transient and dose-dependent inhibition of the growth of these beneficial bacteria occurred [36]. Another study supporting these findings evaluates the cytotoxic effects of AgNPs on human intestinal cells after in vitro digestion. While the physicochemical properties of AgNPs changed during oral-gastric-intestinal fluid digestion, overall toxicity was largely preserved. It was observed that digested commercial AgNPs reduced cell viability, increased ROS production, but did not induce apoptosis. Hydroxyethyl cellulose-coated AgNPs showed lower toxicity, while polymer-coated commercial AgNPs showed moderate toxicity [37]. Moreover, pro-inflammatory activities of AgNPs are also known to lead to intestinal toxicity [38]. Therefore, in addition to their potent activities, the toxicity of AgNPs and how they affect organs and tissues vital to human health should be comprehensively tested; the long-term and dose-dependent effects of AgNPs should be evaluated on 3D co-cultures and advanced models [29].

In conclusion, the cytotoxicity and anticancer effects of AgNPs are closely related to their synthesis methods and physicochemical properties such as size, shape, and surface chemistry. These properties shape both the toxicity mechanisms and the targeted anticancer responses. Given that the anticancer activity of AgNPs is also largely dependent on their physicochemical properties, a focused discussion of how synthesis methods, size, and shape modulate these effects is presented in the following section. While cytotoxicity and anticancer effects share common pathways, they differ in context and selectivity, highlighting both the potential risks and therapeutic value of AgNPs. Therefore, these dual effects must be carefully evaluated for the biomedical applications. This bidirectional effect becomes even more critical, especially with the understanding of the unique mechanisms that occur in cancer cells.

## 3. Anticancer Activity of AgNPs

NPs are widely utilized in cancer therapy for both targeted delivery and cytotoxicity, and they also exhibit broad biomedical applications [39]. This section discusses the various mechanisms underlying the anticancer activity of AgNPs, which are considered among the most promising nanomaterials in cancer research.

### 3.1. Anticancer Mechanisms of AgNPs

**Figure 2:**
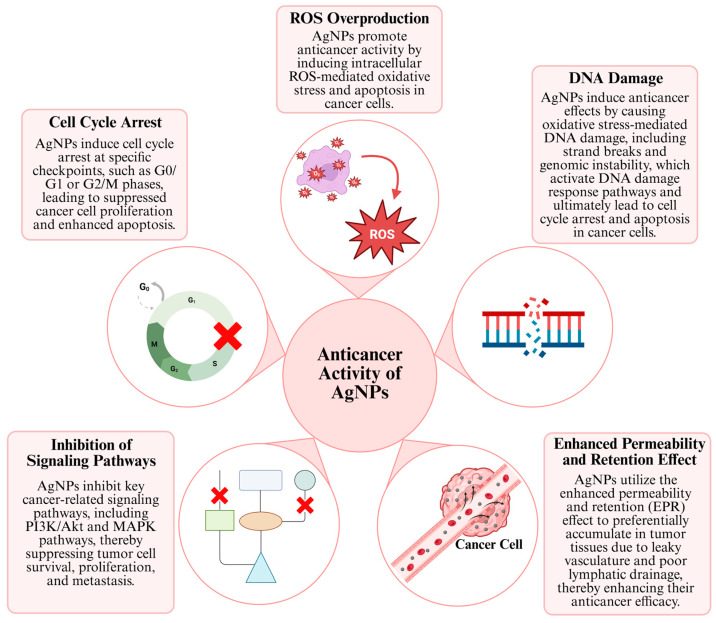
Anticancer mechanisms of AgNPs [29,30,31].

One of the most important reasons why AgNPs are promising in anticancer research is their ability to exhibit specific anticancer activity against various cancer cell lines, while demonstrating reduced toxicity towards healthy cells. This property makes AgNPs an important alternative agent for cancer treatments, specifically in lung and breast cancer [40]. The anticancer effect mechanisms of AgNP generally consist of concepts such as inducing apoptosis or autophagy by stimulating signaling pathways, affecting the cell cycle, increasing DNA damage and ROS production by stimulating and affecting the enhanced permeability and retention (EPR) effect (Figure 2) [41].

AgNPs modulate various intracellular signaling pathways that regulate cell survival, apoptosis, and proliferation. Depending on cellular context, they can suppress oncogenic pathways or activate tumor-suppressor pathways, thereby inducing controlled cell death in cancer cells. Inhibition of certain cascades, such as PI3K, AKT, MAPK, and mTOR, suppresses downstream pro-survival signaling and reduces the expression or activity of associated targets, including BCL2 and HIF-1α, thereby impairing tumor cell survival [8,42,43]. At the same time, activation of the caspase pathways, such as caspase-3 and caspase-9, triggers apoptosis through ROS production and mitochondrial dysfunction via the release of cytochrome c. This dual regulation forms the basis of the anticancer effect of AgNPs [44,45]. Some studies have observed that stimulating signaling pathways triggers apoptosis in cells, particularly cancer cells.

For example, one study reported that 13 nm AgNPs induced apoptosis in A549 lung cancer cells. This effect was associated with suppression of the NF-κB pathway, increased expression of pro-apoptotic proteins (Bax and Bad), and decreased levels of the anti-apoptotic protein Bcl-2 [32]. In another study, AgNPs measuring 8–22 nm were synthesized using *Citrus maxima* peel extract and tested on H1299 lung cancer cells. These NPs also induced apoptosis by inhibiting the NF-κB pathway and regulating caspase-3 and surviving levels. As a result, cellular growth was suppressed in H1299 cells with apoptosis induction, including the suppression of H1299 tumors in SCID mice [46].

Furthermore, the anticancer effects of AgNPs are not limited to suppressing signaling pathways; it also occurs through the excessive production of ROS and the resulting DNA damage. In this process, after being taken up into the cell via phagocytosis or endocytosis, AgNPs or ionized Ag^+^ species translocate to redox-active organelles, particularly mitochondria, where they induce excessive ROS production. This ROS accumulation disrupts intracellular redox balance by suppressing glutathione levels and associated antioxidant enzymes, increasing oxidative stress. Simultaneously, mitochondrial membrane integrity is compromised by the downregulation of Bcl-2 and the upregulation of Bax; this facilitates cytochrome c release and the activation of caspase-9 and caspase-3, ultimately leading to programmed cell death [47]. When AgNPs cause DNA damage through ROS production in cells, they increase p53 protein and upregulate p21 expression, which in turn increases CDK-cyclin complexes, leading to the induction of apoptosis [48]. This resulting imbalance leads to impaired mitochondrial function and impaired energy metabolism. Furthermore, structural damage occurs in DNA, which exceeds the cells’ repair capacity and triggers apoptosis [49].

A mechanistic study observed that AgNPs applied to MCF-7 and HCT-116 (colorectal cancer cell line) cells for 24 h increased ROS production by approximately 150 to 250%. They also emphasized that ROS affects apoptosis. They also demonstrated that AgNPs induce apoptosis in cancer cells through the p53/bax/bcl-2 and caspase-3 pathways [50]. Another study investigated the anticancer mechanisms of poly(N-vinyl pyrrolidone/chitosan/AgNP) nanocomposites. While increases in MDA and H_2_O_2_ were observed following nanocomposite application, CAT activity was decreased. This was thought to trigger lipid peroxidation in the cell membrane, leading to structural damage. Furthermore, the Bcl-2 gene was reported to be suppressed, while the BAX gene was significantly increased. Subsequently, Caspase-3 and p53 proteins were observed to increase by 203% and 109%, respectively [51]. In conclusion, ROS production and its mechanism of action on cancer cells are promising in systems developed for use in anticancer activity and serve as the key to strong activity.

Another mechanism underlying the anticancer activity of AgNPs is the EPR effect. EPR is commonly defined in the literature as a unique biological phenomenon observed in tumor tissues. Large molecules or NPs (e.g., albumin, polymer-bound drugs, liposomes, nanodrugs) accumulate more readily at the tumor site and persist there longer than in normal tissues due to leaky vasculature and poor lymphatic drainage [52,53]. Although the EPR effect can be used for drug delivery systems, the irregular distribution of vascular spaces can be a limiting factor in treating tumors [54]. Another limiting feature of the EPR effect is that factors such as vascular permeability of tumors, blood flow, tumor type, and location can affect EPR efficiency, which cannot provide a comprehensive treatment [8,55].

A study conducted to investigate the anticancer activity of epirubicin-coated AgNPs tested the synergistic effects of AgNPs with the chemotherapeutic agent epirubicin. In this study, the researchers attributed the specific local effects of AgNPs to the EPR effect, while epirubicin reduced AgNPs and controlled their release into tumor tissue [56,57]. Another study by Veeragoni et al. examined the antitumor effects of chemically and green-synthesized AgNPs against different cancer cell lines in vitro and in vivo. Findings showed that green-synthesized AgNPs are more stable, release ions preferentially at acidic tumor pH, and exhibit better selectivity. Besides that, chemically synthesized AgNPs showed greater toxicity to normal cells, with stronger genotoxic effects, and researchers have attributed these effects to the EPR effect in green-synthesized AgNPs. However, green-synthesized AgNPs were found to have better biocompatibility and exhibited potent anticancer activity against B16-F10 cells and in an in vivo melanoma cancer model in C57BL/6 mice [58].

Among the mechanisms underlying the anticancer effects of AgNPs, cell cycle arrest is of great importance and can generally be defined as a termination in the G0/G1 or G2/M phases and the associated mitochondrial damage [59]. It has been reported that AgNPs induce cell cycle arrest in cells of common cancer types such as colon, lung, and breast cancer, and that this effect occurs through the downregulation of cyclin and CDK (cyclin-dependent kinases) proteins and the upregulation of p21 and p53, along with ROS production [48]. The cell cycle process is controlled by CDKs. AgNPs suppress the expression of these enzymes and block the transition between different phases of the cell cycle in these cells [60]. As a result of these biochemical processes, cancer cells exposed to AgNPs stop proliferating, those unable to repair their DNA undergo apoptosis, or lose their proliferative capacity and enter the death process. Therefore, in addition to inducing cell death, AgNPs contribute to suppressing tumor growth by strategically halting the cell cycle [48].

However, it should be known that while AgNPs have an anticancer effect, all these mechanisms are simultaneously active. For instance, Vahabirad et al. examined the molecular mechanisms underlying the anticancer activity of AgNPs against SKBR3 breast cancer cells. Reported significant inhibition of the PI3K/AKT signaling pathway, which plays a key role in cell proliferation. Lower expression of PI3K and AKT genes diminished the survival signals in cells, which led to slower growth and reduced viability of tumor cells. In addition, the AgNPs caused ROS production, as mentioned earlier, which produced a distinct oxidative stress response characterized by an increase in total oxidant status (TOS) and malondialdehyde (MDA) levels and a decrease in total antioxidant capacity (TAC). This oxidative imbalance reduced the cellular antioxidant defensive mechanism, making cells more vulnerable to apoptosis. In parallel, the increase in Bax gene expression and the rise in caspase-3/7 enzyme activity, together with the suppression of the anti-apoptotic Bcl-2 gene, confirmed that AgNPs triggered the internal apoptosis pathway through inhibition of the PI3K/AKT pathway, thereby facilitating programmed cell death. In conclusion, these findings reveal that AgNPs exert their anticancer effects through a multifaceted mechanism involving oxidative stress induction, signaling pathway activation, and suppression of PI3K/AKT-mediated survival signaling networks [61].

In the other study conducted by Srisaisap et al., the N-terminal truncated PS2Aa1 toxin was integrated into the produced MOEAgNPs, AgNPs that were biosynthesized from *Moringa oleifera* leaf extract, and their anticancer efficacy and activation mechanisms were investigated. To this end, the activity of PS2-MOEAgNPs treated with maltose-binding protein (MBP) was tested against T-cell leukemia, MOLT-4, and Jurkat cell lines. In Jurkat and MOLT-4, intracellular vacuolations, nuclear shrinkage, and cell lysis were observed; these are morphological indicators of late apoptosis and necrosis. The parasporin-2 protein (PS2Aa1) causes cell death by creating pores in the cancer cell membrane, disrupting ion balance, and damaging the cytoskeletal structure and organelles. This mechanism remained active in PS2-MOEAgNPs. Furthermore, DNA damage caused by ROS production induced by AgNPs was observed to be greater in PS2-MOEAgNPs than in other experimental groups, MOEAgNPs, and MBP-tPS2. Considering that Parasporin-2 is known to cause apoptosis through caspase activation and regulation of the AKT pathway, it has been suggested that this may also contribute to the cytotoxic effect of PS2-MOEAGNPs. Finally, MOEAgNPs demonstrated a high uptake capacity in cancer cells, facilitating the transport of the MBP-tPS2 protein into the cell, thereby enhancing the specific delivery and efficacy of the toxin to the cell [62]. As a result, studies have shown that multiple mechanisms are involved simultaneously in the anticancer effects of AgNPs.

Beyond their anticancer activity through diverse mechanisms, AgNPs actively modulate the cellular microenvironment through cytokines and immune cell behavior. These effects extend to both the tumor microenvironment (TME) and immune system components, influencing inflammatory signaling, immune cell recruitment, and antitumor immune response [63]. Understanding these interactions is essential for evaluating the therapeutic potential and safety of AgNP-based anticancer strategies. Cancer biology is genetically complex and highly variable; therefore, attempting to understand it from a single perspective is flawed, as it is a phenomenon that requires analysis from many different angles. One reason why AgNPs show promise as anticancer agents is their ability to respond to this complex microenvironment [47].

Studies have shown that AgNPs do not directly activate the immune system in a lethal manner; instead, they reprogram the TME by regulating the cytokine network [64]. TME is tumor-strengthening because it makes it more difficult for the immune system to detect the details of the tumor. Cytokines derived from TME are known to increase the expression of inhibitory checkpoint molecules in T cells, such as PD-1 and CTLA-4 [65]. Immune checkpoint inhibitors, such as anti-PD1 and anti-CTLA antibodies, have been observed to show promise in anticancer approaches by reversing immunosuppression and enabling the immune system to work against the tumor [66]. In the studies conducted, the levels of TNF-α and IL-6 cytokines, which are normally expected to increase in parallel with tumor growth, remained at normal levels in cancer cells treated with AgNPs coated with mouse serum albumin (MSA). This indicates less inflammation and a smaller tumor size. This has been attributed to the optimization of the behavior of AgNP-MSA in the tumor microenvironment [67]. Furthermore, the effect of AgNPs on VEGF and SDF-1α expression demonstrates that angiogenesis and stromal remodeling can be regulated in TME. VEGF is one of the key regulators of tumor progression by increasing nutrient and oxygen supply, while SDF-1α is a critical chemokine that directs the migration of stromal and progenitor cells to the tumor site. The modulation of these factors by AgNPs may contribute to the shaping of the cell migration, vessel formation, and stromal-tumor cell interactions in the TME. This further demonstrates that AgNPs can also be considered not only cytotoxic but also regulatory agents targeting the microenvironment [68]. Additionally, the local inflammation and vascular damage induced by AgNPs in the tumor microenvironment trigger intense macrophage infiltration into the tumor site, thereby reorganizing immune cell dynamics. This process leads to a disruption of the dominance of immunosuppressive M2-like tumor-associated macrophages (TAMs) and the redirection of macrophages towards the antitumor M1 phenotype. The reduction in myeloid-derived suppressor cell (MDSC) population by AgNP-modified systems creates a more favorable immune microenvironment that supports the sustainability of this M2 → M1 polarization. The increase in pro-inflammatory cytokines such as TNF-α and IL-6 indicates that an M1-dominant response is strengthened, which is critical for the efficacy of antitumor immunity [69].

### 3.2. Influence of Synthesis Methods and Physicochemical Properties on the Anticancer Activity of AgNPs

In addition to the mechanisms that provide anticancer activity of AgNPs, it is known that the synthesis method and, indirectly, the shape, size, and surface chemistry of AgNPs directly affect this anticancer activity. These physicochemical properties can be controlled by synthesis methods, enabling AgNPs to perform specific tasks. Therefore, when discussing the anticancer activity of AgNPs, it is necessary to emphasize the importance of synthesis methods and physicochemical properties.

AgNPs can be synthesized using a variety of approaches, broadly classified as chemical, physical, and green synthesis methods. Chemical synthesis typically involves the reduction of silver salts using chemical reducing agents and stabilizers, allowing precise control over particle size and shape, but may introduce toxic residues [70,71]. Physical methods, such as laser ablation, irradiation, evaporation-condensation, and lithography, generally produce highly pure NPs, although they often require specialized equipment and high energy requirements [72]. In contrast, biological or green synthesis methods employ plant extracts, microorganisms, or biomolecules as reducing and capping agents, offering an environmentally friendly alternative with enhanced biocompatibility; however, variations in the biological source can lead to distinct physicochemical properties and biological responses, resulting in different anticancer outcomes [73]. These synthesis strategies directly influence the physicochemical characteristics of AgNPs, which in turn modulate their biological properties, particularly anticancer activities and their associated mechanisms of action (Table 1).

For instance, in comparative studies, Nayak et al. synthesized AgNPs using extracts from *Cucurbita maxima*, *Moringa oleifera*, and *Acorus calamus* and evaluated their cytotoxic effects against A431 epidermoid carcinoma cells. Among the formulations tested, *A. calamus*-mediated AgNPs showed the most potent antiproliferative activity, with a lower IC_50_ value (~78.6 μg/mL) compared to AgNPs synthesized from *C. maxima* and *M. oleifera*. More importantly, these biosynthesized AgNPs exhibited favorable physicochemical properties associated with improved biocompatibility, such as controlled cytotoxicity, stable zeta potential, and nanoscale size distribution [74]. A direct comparison between green and chemically synthesized AgNPs performed by Balaji et al. reported that plant-mediated AgNPs synthesized using Sida acuta leaf extract exhibited stronger cytotoxic effects against MCF-7 breast cancer cells, with a lower IC_50_ value (100 μg/mL) compared to chemically synthesized AgNPs (132 μg/mL). This enhanced anticancer efficacy was associated with smaller particle size and phytochemical surface capping, which may promote improved cellular uptake and apoptosis [75]. In a study conducted by Kummara et al., green AgNPs reduced cell viability by 2% in lung cancer NCI-H460 cells, while chemically synthesized AgNPs at the same dose showed no significant inhibition. In contrast, chemically synthesized AgNPs caused significant toxicity and increased ROS in normal HDFa cells, while green synthesized AgNPs showed minimal toxicity in normal cells, demonstrating a safer profile [22]. Overall, studies show that when comparing the anticancer activity of differently synthesized AgNPs, green-synthesized AgNPs have the potential to have the highest therapeutic efficacy in anticancer studies due to their flavonoid, polyphenol, and protein content and their greater selectivity compared to other synthesis methods [76].

AgNPs owe their biological activity, such as their anticancer properties, to different physicochemical characteristics, such as size, shape, and surface chemistry and charge. AgNP size stands out as a key parameter determining the mode of interaction with the cell membrane, cellular uptake mechanisms, surface area/volume ratio, and ion release kinetics in the biological environment [77,78]. Accordingly, smaller AgNPs possess a higher specific surface area than larger counterparts, which facilitates enhanced Ag^+^ ion release, leading to disruption of cellular metabolic pathways and induction of DNA damage in cancer cells [79].

As an example of the size-dependent anticancer effect of AgNPs, the study conducted by Islam et al. showed that AW60-AgNPs (acid-treated W60-AgNPs; 12.82 ± 4.83 nm in size) exhibited the highest dose- and time-dependent anticancer efficacy against K562 leukemia cells when compared to other AgNP samples. When administered at a dose of 6 μg/mL, AW60-AgNPs produced the strongest apoptotic response with 64.7% early apoptosis and 25.29% late apoptosis rates; These values were found to be significantly higher than the early and late apoptosis rates observed in larger AgNPs, namely RT-AgNPs (ambient temperature synthesis; 35.04 ± 11.78 nm in size) with 28.6%, W60-AgNPs (water-bath synthesis at 60 °C; 31.71 ± 7.04 nm in size) with 52.5%, early and late apoptosis rates with 16.7%, and W70-AgNPs (water-bath synthesis at 70 °C; 29.58 ± 6.83 nm in size) with 45.5%, early and late apoptosis rates with 14.4% [79]. Another study demonstrated that ultrasmall (<5 nm) AgNPs exhibited high and uncontrolled toxicity, failing to distinguish between cancer and healthy cells. In contrast, approximately 40 nm in size and negatively charged (Ag@TSC2) NPs generated potent cytotoxicity in cancer cells through increased intracellular accumulation and ROS-mediated apoptosis, while showing no significant toxicity in healthy cells. This selective anticancer effect has been consistently confirmed in 2D cell cultures, 3D tumor spheroids, and hiPSC-derived colon cancer organoids. Furthermore, Ag@TSC2 treatment significantly suppressed tumor growth in the in vivo CT26 tumor model [33]. The study by Gołuński et al. also showed that AgNPs directly interact with doxorubicin, and this interaction significantly alters anticancer efficacy depending on the NP size. 50 nm AgNPs, in combination with doxorubicin, significantly reduced cell viability in SKBR3 and MDA-MB-231 breast cancer cells. In contrast, the effect of 5 nm AgNPs was more limited and contributed less to anticancer activity. In conclusion, this study observed that AgNP size affects not only its own biological activity but also the efficacy of its use with chemotherapeutic drugs with which it exhibits a synergistic effect [80].

For the biological activities of AgNPs, their shape is just as important as their size. While the shape of AgNPs is highly variable, some examples include spherical, triangular, rod-like, cube, and polygonal AgNPs [81]. Furthermore, studies have shown that altering the shape of AgNPs can also change their stability and toxic properties [82,83]. Yin et al. demonstrated that the anticancer efficacy of AgNPs varied significantly depending on the NP shape. Triangular AgNPs (tAgNPs) exhibited higher cytotoxicity in SKOV3 ovarian cancer cells compared to spherical forms. The study showed that tAgNPs increased ROS production in cells, consequently triggering caspase-3 activation and inducing apoptosis through cell cycle arrest in the G0/G1 phase [84]. Additionally, one study showed that the cellular uptake and dissolution of triangular silver nanoprisms and spherical silver NPs were compared in hMSC and HaCaT cells. Nanoprisms showed limited dissolution in pure water, but rapid dissolution under acidic pH and isotonic conditions; their tips rounded quickly due to high surface energy. Spherical AgNPs, on the other hand, dissolved largely within 24 h. In both cell types, the NPs lost more than 90% of their volume after 24 h. In terms of cellular uptake, hMSCs internalized nanoprisms more than spherical AgNPs, while no shape-dependent difference was observed in HaCaT cells. This was explained by the more flexible membranes of hMSCs and the larger contact area created by plate-like nanoprisms [85]. In conclusion, these findings reveal that AgNP size and its associated effective morphology are key parameters that critically modulate anticancer efficacy.

On the other hand, surface chemistry in AgNPs is an important physicochemical parameter that directly modulates toxicity, biocompatibility, and anticancer efficacy by determining the stability of NP in the biological environment, their cellular interactions, Ag^+^ ion release, and protein corona formation [86,87]. In a study by Barbalinardo et al., citrate-coated AgNPs (AgNPs-cit) significantly reduced cell viability and induced morphological changes suggestive of programmed cell death in microsatellite instability (MSI-H) LoVo colorectal cancer cells. In contrast, EG_6_OH-coated AgNPs (AgNPs-EG_6_OH) showed minimal cytotoxic effects in both cancer cells and healthy colonocytes. These findings highlight that the surface chemistry of AgNPs is a critical design parameter for achieving targeted and safe anticancer effects by determining cellular interactions and intracellular distribution [88]. Another study by Moors et al. also showed that PVP- and BSA-coated AgNPs were compared with DPPC/cholesterol-based liposomal AgNPs in MCF-7 breast cancer cells. While BSA-coated AgNPs showed the highest cytotoxic effect even at low doses (IC_50_ = 2.5 μL/mL), PVP-coated and liposomal AgNPs exhibited higher IC_50_ values, and it was shown that liposomal encapsulation reduced cytotoxicity by controlling the entry of NPs into the cell [89].

**Table 1:** Synthesis methods and action mechanisms of AgNPs used in current anticancer studies.

Cancer Type	Synthesis Method	Mechanism of Action	Reference
- Murine Colon Carcinoma (CT26) - Murine Mammary Gland Carcinoma (4T1)	- Chemical synthesis, - Surface functionalization with trisodium citrate (TSC) or (TMA).	- ROS-mediated oxidative stress, - DNA interaction and predominantly late-stage apoptosis, - Cellular uptake and cytotoxicity are strongly dependent on nanoparticle size and surface charge.	[33]
- Breast cancer (MCF-7) - Murine Mammary Adenocarcinoma (EO771)	- Green aqueous co-precipitation synthesis by mixing silver nitrate and sodium nitroprusside at room temperature.	- Selective cytotoxicity via early and late apoptosis associated with nitric oxide (NO) release; inhibition of tumor growth	[90]
- Breast Cancer (MCF-7, MDA-MB-231); - Lung Cancer (KAIMRC-2)	- Plant-mediated green synthesis using *Pulicaria undulata* extract	- Dose-dependent cytotoxic and antiproliferative effects, - In silico–predicted targeting of TNF-α, GPCR-related signaling, and metabolic enzymes (aldose reductase) by plant-derived bioactive compounds.	[91]
- Breast Cancer (MCF-7)	- Fungal-mediated green synthesis of AgNPs using *Aspergillus fumigatiaffinis* filtrate; - Paclitaxel loading to form AgNPs@PTX nanocarrier.	- Enhanced cytotoxicity via apoptosis and necrosis induction, - DNA fragmentation, and G0/G1 cell cycle arrest, - Synergistic effect of paclitaxel delivery by AgNPs leading to increased anticancer efficacy at lower PTX dose.	[92]
- Unspecified Cancer Cell	- Chemical reduction (NaBH_4_) - Green synthesis using gallic acid - seed-mediated growth (to obtain triangular nanoprisms)	- pH-sensitive doxorubicin release, - Enhanced ROS generation due to AgNP partial dissolution and DOX synergy, - Electrostatic interaction with DNA phosphate backbone.	[93]
- Breast Cancer (MCF-7) - Prostate Cancer (DU-145)	- Green biosynthesis via AgNO_3_ reduction using cell-free supernatants of endophytic *Streptomyces* sp. KE4D and *Bacillus safensis* KE4K	- AgNP-induced ROS generation leading to oxidative stress, - Membrane and mitochondrial damage, DNA interaction, - Activation of apoptosis and cell death pathways	[94]
- Gastric Cancer (MKN45, AGS)	- Green synthesis of AGNPs from *Ardisia gigantifolia*.	- Induction of cell cycle arrest at the G0/G1 phase in AGS and MKN45 cells - Suppression of cancer cell migration, as shown by the wound-healing assay - Induction of cellular senescence, evidenced by increased SA-β-gal-positive cells - Significant increase in intracellular ROS generation	[95]
- Breast Cancer (MCF-7, MDA-MB-231)	- Commercially available PVP-coated AgNPs combined with tamoxifen	- Strong intracellular ROS overproduction leading to oxidative stress - Activation of apoptosis as the primary mode of cell death (early and late apoptosis) - Upregulation of proapoptotic and oxidative stress–related genes (p53, Bax, sod-1) - Downregulation of antiapoptotic gene Bcl-2 - DNA damage induction remaining below the accepted genotoxicity threshold - Inhibition of cell migration and colony formation, particularly in highly metastatic MDA-MB-231 cells	[96]
- Hepatic Cancer (HepG2)	- Green synthesized AgNPs from *Lactobacillus acidophilus*.	- Induction of oxidative stress through increased ROS production - Mitochondrial dysfunction and disruption of mitochondrial membrane potential - Activation of intrinsic apoptosis evidenced by caspase activation - Upregulation of AMPK expression associated with autophagy initiation - Suppression of mTOR signaling leading to reduced cell proliferation - Downregulation of anti-apoptotic and pro-metastatic genes (BCL-2, MMP-9, α-SMA) - Enhanced secretion of pro-inflammatory cytokines (TNF-α, IL-33) contributing to immune-mediated anticancer effects	[97]
- Breast Cancer (MCF-7)	- Green synthesis of AgNPs via reduction of AgNO_3_ using aqueous leaf extract of *Indigofera heterantha*.	- Excessive intracellular ROS generation leading to oxidative stress, - Disruption of cell membrane integrity and increased permeability, - Nuclear condensation, fragmentation, and chromatin damage, - Activation of apoptotic cell death confirmed by AO/EB, DAPI, and Annexin V/PI assays, - Mitochondrial dysfunction and lipid peroxidation, - Loss of p53 and oxidative DNA damage.	[98]
- Gastric Cancer (AGS)	- Green synthesis of AgNPs from *Caralluma pauciflora*.	- Excessive intracellular ROS generation confirmed by H2DCFDA fluorescence, - Morphological alterations, including cell shrinkage and membrane blebbing, - Induction of apoptotic cell death confirmed by AO/EB staining and DNA fragmentation, - Downregulation of PI3K/AKT/mTOR signaling pathway, - Suppression of survival and proliferation signaling leading to apoptosis-mediated tumor cell death.	[99]
- Prostate cancer (PC3; 2D monolayer and 3D tumor spheroids)	- Green synthesized AgNPs	- Induction of intracellular ROS following AgNP internalization - Mitochondrial membrane depolarization indicated by decreased Rh123 fluorescence - Cell cycle arrest with increased sub-G1 population reflecting apoptotic DNA fragmentation - Nuclear condensation and early apoptotic morphology confirmed by AO/EtBr staining - DNA fragmentation consistent with apoptosis-mediated cell death	[100]
- Breast Cancer (MCF-7)	- Green synthesis of AgNPs from *Trigonella foenum-graecum*	- Suppression of TNF-α expression at both mRNA and protein levels in MCF-7 cells - Anti-inflammatory activity through downregulation of TNF-α-mediated signaling pathways - Controlled intracellular release of Ag^0^ ions contributing to biological activity without marked cytotoxicity	[101]
- Human Pancreatic Cancer (PANC-1, AsPC-1, MIA PaCa-2)	- Green synthesis of AgNPs from *Zingiber officinale leaf*	- AgNPs and Ag^+^ ions modulate cellular redox balance through scavenging of reactive ROS and reactive nitrogen species (RNS) - Ag^+^ ions bind to DNA and cellular proteins, leading to disruption of essential cellular functions - Small-sized AgNPs are capable of crossing the cell membrane, resulting in more efficient uptake by tumor cells.	[102]

The modifiability of AgNPs extends beyond their cytotoxicity, giving them a strong potential as anticancer agents. This potential is realized through synthesis methods that both increase their stability and allow for the adjustment of their optimal shape and size. Green synthesis approaches, in particular, utilize plant-based polyphenols and flavonoids to reduce AgNP toxicity to healthy tissues while maintaining their toxicity to cancer cells. Biocompatibility also highlights the strengths of green synthesis. However, the difficulty in standardizing green synthesis, the uncertainty surrounding the molecules involved in reduction and stabilization processes, and the limited control of physicochemical properties can lead to the preference for other methods, especially chemical synthesis methods.

## 4. Anticancer Studies of AgNPs

While AgNPs and AgNP-based systems hold high potential in anticancer studies, the number of studies conducted in this area is relatively small and should be expanded. This section provides AgNP-based treatment methods developed in vivo and in vitro in recent years. A review of the literature indicates that most studies have focused on specific cancer cell lines, and these studies have been categorized accordingly. As shown in the subheadings, different methods have been tested on cancer cell lines associated with different organs, and the results of these methods have been used to evaluate the anticancer potential of AgNPs. Furthermore, as shown in Table 2, there has been increasing research on anticancer properties of AgNPs in recent years. These studies, which aim to utilize the different physicochemical properties of AgNPs, contribute to the future potential of AgNPs as anticancer agents.

### 4.1. Breast Cancer

Rimbu et al. aimed to evaluate the antitumor properties of *Taraxacum officinale* (D-Dandelion) and *Artemisia annua* (SW-sweet wormwood) extracts, as well as their efficiency as bioreducing agents in the green synthesis of AgNPs for potential therapeutic applications [120]. SEM/TEM analyses revealed circular, triangular, and rod-shaped particles had been synthesized. Antioxidant (DPPH) and antimicrobial tests were conducted before anticancer activity tests. AgNP–Eaq–SW was found to be the most antioxidant active (83.1%), while lower activity was observed in Dandelion derivatives. In the anticancer activity tests, different cancer cell lines (e.g., MDA-MB-231, LoVo, HepG2) and normal human endothelial cells (HUVEC) were compared. In these tests using the MTS assay, AgNPsE_ETOH_3%-D demonstrated high cytotoxicity, particularly in colon (LoVo) and breast cancer (MDA-MB-231) cells. Because these AgNPs were prepared with a lower ethanol content, they consisted of smaller particles, resulting in a more homogeneous distribution and, therefore, more effective intracellular effects. Furthermore, while the synthesized AgNPs did not appear to reduce the overall viability of the HUVEC line, the presence of toxic effects on healthy cells was confirmed. This study highlights the different biological properties of plant-based NPs, especially their anticancer and antioxidant properties.

Alghamdi et al. also tested the anticancer activity of AgNPs synthesized from three different plants [91]. Characterization studies of the synthesized AgNPs revealed that R.S.-AgNPs and C.P.-AgNPs were spherical, while C.C.-AgNPs had a triangular structure. For the test of anticancer activity, MDA-MB-231 (Triple Negative Breast Cancer-TNBC), MCF-7, KAIMRC-2 (patient-derived breast cancer line), and MCF-10A (non-tumorigenic epithelial cell) cell lines were used. As a result, C.P.-AgNPs were determined as the most potent anticancer agent with an IC_50_ value of 121.2 µg/mL in the MCF-7 cell line. R.S.-AgNPs were also the most potent agent with a value of 154.7 µg/mL in MDA-MB-231 cells. The AgNP that provided the highest inhibition in the KAIMRC-2 line was C.C.-AgNPs (IC_50_ = 135.1 µg/mL). In contrast, IC_50_ values in the MCF-10A cell line ranged from 303–460 µg/mL, indicating that it is less toxic to normal cells than to tumor cells. Additionally, Mitoxantrone was tested as a positive control group to obtain comparative data. Mitoxantrone’s IC_50_ values were measured as ~1–2 µg/mL, indicating that it is a more potent anticancer agent, but it was also observed to have a highly toxic effect on MCF-10A. Consequently, all AgNPs exhibited dose-dependent cytotoxicity, producing significant inhibition in cancer cells. Thus, AgNPs synthesized from three different plant extracts were found to exhibit significant cytotoxic effects against breast cancer cell lines and also have potential as safe oral anticancer agents with their low toxicity and selective effects.

Aboul-Nasr et al. used the AgNPs in this study that were synthesized using the filtrate of the fungus paclitaxel (PTX) [92]. The AgNPs@PTX nanocarrier was prepared by combining biosynthetically obtained AgNPs with PTX. TEM analyses determined that AgNPs had an average size of 14 nm, and AgNPs@PTX particles had an average size of approximately 28 nm. The cytotoxic effects of AgNP and AgNPs@PTX formulations on MCF-7 breast cancer cells were evaluated by MTT assay, and IC_50_ values were calculated as 15.47 µg/mL for AgNPs and 1.7 µg/mL for AgNPs@PTX, respectively. It was determined that AgNPs@PTX exhibited significantly higher cytotoxicity compared to AgNPs. The apoptosis-inducing effects of AgNPs and AgNPs@PTX nanocarriers in MCF-7 cells were evaluated by Annexin-V FITC/PI staining. AgNP application significantly increased apoptosis, while the AgNPs@PTX formulation further strengthened this effect. Cell cycle analyses showed that both formulations induced changes in DNA content and stopped in the G0/G1 phase. A statistically significant increase was observed in the G0/G1 phase compared to the control group (AgNP: 74.5%; AgNPs@PTX: 82.3%; control: 61.7%), indicating that cell proliferation was suppressed. Morphological analyses showed that AgNP-treated MCF-7 cells exhibited signs of apoptosis, such as reduction in cell size, breakdown of the nucleus, and condensation of chromatin. On the other hand, treatment with AgNPs@PTX resulted in necrotic changes characterized by cell swelling and membrane rupture. Mechanistic analyses revealed that the nanocarrier exhibited a potent therapeutic effect by simultaneously inducing both apoptosis and DNA fragmentation. In summary, the developed AgNPs@PTX nanocarrier exhibited five-fold higher anticancer activity compared to bare AgNPs and significantly reduced toxicity to healthy cells.

Another study to measure the anticancer activity of AgNPs was conducted by Abdel-Kareem et al. [121]. In this study, *A. templicola MAK 223* extract was used for the first time for the synthesis of green AgNPs. The synthesized AgNPs were then subjected to characterization tests. In these tests, AgNP formation was confirmed by UV-Vis, and TEM images revealed spherical AgNPs with an average diameter of 17.8 nm. DLS results also demonstrated the high stability of these AgNPs. Cell cycle distribution following AgNP application in MCF-7 cells was analyzed based on fluorescence intensity. After 48 h of incubation with AgNPs, a significant decrease in cell viability was observed, and the IC_50_ value was determined to be 50 µg/mL. Compared to the control group, AgNP-treated cells exhibited a decrease in the S and G2/M phases, cell cycle arrest, and induction of apoptosis. Additionally, AgNPs were determined to induce apoptosis in MCF-7 cells through the suppression of MMP-9. The study confirmed that fungal-derived AgNPs exhibit anticancer activity through the inhibition of apoptosis, DNA damage, and cell migration. While this demonstrates the cytotoxic effect of AgNPs produced using green methods against cancer cells, measuring the cytotoxicity of the synthesized AgNPs in a normal/healthy cell line would have been more appropriate for obtaining comparative data.

Vimalanathan et al. investigated the drug delivery potential of AgNPs [122]. AgNP-integrated nanocomposites were formed with graphene oxide (GO) and reduced graphene oxide (rGO) powders. Furthermore, SEM images revealed that AgNPs were distributed homogeneously and spherically on the nanosheet surface. The obtained GO, rGO, GO-AgNP, and rGO-AgNP samples were used to treat the MCF-7 cell line and measure the cytotoxicity of the AgNPs and GOs. GO application caused a significant decrease in cell viability, especially from 1 µM onwards, with the viability rate decreasing to 68.7% at 50 µM concentration. The GO-AgNP combination showed a stronger effect, causing a 58.7% decrease in viability at the same dose. This effect was attributed to the anticancer properties of AgNPs related to oxidative stress. While rGO alone reduced cell viability by 70.1% at 50 µM, AgNP application alone showed 82% cytotoxicity. However, the rGO-AgNP combination reduced cell viability to 65%, revealing a more pronounced synergistic effect between the two materials. Additionally, the effect of GO, rGO, GO-AgNP, and rGO-AgNP on macrosphere formation in MCF-7 cells was also measured, and the results showed that all samples had a negative effect on macrosphere formation. This decrease was particularly pronounced in cells treated with rGO and rGO-AgNP compared to GO and GO-AgNP applications. Additionally, researchers found that GO-AgNP and rGO-AgNP structures significantly reduced the expression of stem cell marker genes in MCF-7 breast cancer cells; however, AgNP alone had no effect on the Nanog gene. To sum up, these results indicate that rGO and rGO-AgNP structures suppress the formation of tumor stem cells and that their anticancer activity not only induces cell death but also inhibits the formation of new colonies.

Another study evaluating the anticancer activity of AgNPs was conducted by Darvish et al. [123]. In this study, AgNPs were synthesized using an aqueous extract of *Ducrosia anethifolia*, and the formation of spherical AgNPs with an average size of 9.41 nm was confirmed by UV-Vis, TEM, and DLS analyses. The cytotoxic effects of the synthesized AgNPs were investigated in MCF-7 and MDA-MB-231 breast cancer cell lines using the MTT test, and a significant dose and time-dependent decrease in cell viability was observed. After 48 hours of application, the IC_50_ values were determined as 36.56 µg/mL for MCF-7 cells and 19.87 µg/mL for MDA-MB-231 cells, indicating that MDA-MB-231 cells were more sensitive to AgNPs. Gene expression analyses also revealed that AgNP application induced apoptosis by increasing pro-apoptotic genes such as Bax and Bad while suppressing anti-apoptotic genes such as Bcl-2 and c-FLIP, thus confirming the anticancer potential of plant-derived green synthesized AgNPs.

In conclusion, AgNP-based nanomaterials showed significant cytotoxic and anti-proliferative effects, generally on the MCF-7 and MDA-MB-231 cell lines, through mechanisms such as apoptosis induction via various ways and cell cycle irregularities; however, further research is needed to clarify their selective cytotoxicity and long-term biosafety of AgNPs.

### 4.2. Lung Cancer

Pandiarajan et al. interpreted and tested the anticancer activity of AgNPs from a different perspective [124]. For this reason, the synthesis of AgNPs was carried out using *Nigella sativa* extract. It was suggested that coating the surfaces of *Lactobacillus* bacteria with NS-AgNPs would allow cancer cells to more readily take up L-NS-AgNPs. Afterwards, characterization tests were performed on the AgNPs, and DLS and SEM analyses showed that the NS-AgNPs formed spherical clusters with an average size of 7 nm. L-NS-AgNPs demonstrated their anticancer activity against A549 cells in a dose-dependent manner. When the highest concentration of 3 μg/mL was applied, the maximum cell death rate was found to be 54%. The LC_50_ value of these NPs for A549 cells was calculated as 3.005 μg/mL. Moreover, L-NS-AgNPs dose-dependently and significantly killed DLA cells compared to the control group, even within a short treatment time of 4 h. The maximum cell death rate observed following treatment with 3 μg/mL L-NS-AgNPs was 58%. In addition, the safety of L-NS-AgNPs in a living organism was tested in zebrafish embryos, and no apparent abnormalities were observed in embryos exposed to L-NS-AgNPs.

The anticancer activity of Ag@CT NPs synthesized from Clitoria ternatea extract against A549 was investigated by Veetil et al. [125]. In these tests using the MTT assay, the activities of CT and Ag@CT NPs at different doses were tested. The cell viability of CT was 92% at 10 μg/mL and decreased to 50% at 50 μg/mL. When the concentration reached 100 μg/mL, the viability decreased to 3%. The cell viability of Ag@CT NPs at 10 μg/mL was 93% and decreased to 52% at 50 μg/mL. When the concentration reached 100 μg/mL, the viability of Ag@CT NPs decreased to 6%. Subsequently, the IC_50_ values of Ag@CT NPs and CTs were calculated, resulting in values of 51.35 μg/mL and 50.92 μg/mL, respectively. Visual analysis also showed that A549 cells treated with CT and Ag@CT NPs exhibited morphological changes such as aggregation and growth inhibition. Additionally, AO/EB staining analysis of A549 cells revealed that Ag@CT NPs triggered cell death via apoptosis. Typical apoptotic changes, such as chromatin condensation and nuclear fragmentation, were observed during this process. In summary, the results of this study support the potential use of Ag@CT NPs as biocompatible and highly anticancer-active nanotherapeutic agents.

In another study conducted by Raj et al., the anticancer activity of AgNPs synthesized using hesperetin was investigated [126]. For this purpose, MTT assays performed on L929 (fibroblast cell lines) and A549 cell lines revealed that exposure of A549 cells to 25 µL/mL H-AgNPs reduced cell viability to 72%. When the concentration was increased to 100 µL/mL, viability decreased to 59%. Similarly, in L929 cells, viability decreased to 87% and 79% at 25 µL/mL and 100 µL/mL H-AgNP concentrations, respectively. Furthermore, the LD_50_ values for H-AgNPs were calculated as 118.49 µL/mL for A549 and 269.35 µL/mL for A929. These results indicate that H-AgNPs exhibit selective cytotoxicity, an indicator of their anticancer potential. Additional LDH enzyme release assays also highlight the anticancer properties of H-AgNPs. Comprehensive in vitro MTT and LDH assays performed on A549 and L929 cell lines revealed that H-AgNPs possess high anticancer activity and moderate cytotoxicity.

Devendrapandi et al. also tested the anticancer activities of AgNP-CS synthesized from chitosan (CS) [127]. This nanomaterial was tested at different concentrations for 24 h in the A549 cell line. An increase in antiproliferative effect was observed in A549 cells with increasing concentrations of CS-AgNPs. At 70 μg/mL, cell viability decreased by 52%, and this value was determined as the IC_50_. This demonstrated that CS-AgNPs suppressed cell proliferation in a dose-dependent manner. Furthermore, imaging with a fluorescence microscope revealed nuclear morphology alterations, cell shrinkage, and mitochondrial membrane damage. These findings confirmed that CS-AgNPs induce oxidative stress-induced apoptosis; therefore, it was concluded that the nanocomposite exhibits a significant cytotoxic and antiproliferative effect on A549 cancer cells. In summary, the researchers were able to inhibit the proliferation of the A549 cell line with AgNP-CS produced by an innovative synthesis method and confirmed that AgNP-CS exhibits significant cytotoxicity against the cell line.

Awadelkareem et al. tested the anticancer potential of AgNPs synthesized from the leaf extract of the Eruca sativa plant, measuring 8.11 to 15 nm [128]. Using MTT assays, the results showed that the AgNPs developed at different concentrations reduced the viability of A549 cancer cells in a dose-dependent manner. The IC_50_ value was found to be 25.15 µg/mL after 24 h. Scratch and Transwell migration assays were also used to determine the cell migration inhibitory properties of the produced AgNPs. These assays revealed that the AgNPs significantly inhibited migration compared to empty A549 cells. Transwell assays also showed that the applied AgNPs effectively reduced the migration of A549 cancer cells by 18.84% to 91.53%, depending on the concentration. In conclusion, this study not only tested the anticancer properties of AgNPs produced through biosynthesis but also their negative effects on cell migration and demonstrated their anti-metastatic properties.

A study by Wong et al. investigated the effects of treating cisplatin-resistant lung adenocarcinoma with chemically synthesized AgNPs [129]. Spherical and homogeneously distributed AgNPs were first tested in the MTT cytotoxicity test on A549 (cisplatin-sensitive) and A549/DDP (cisplatin-resistant) cells for 24 h. Over this period, the AgNPs exhibited similar cytotoxic activity in both cell lines. They were also found to exhibit dose-dependent cytotoxic activity. Confocal microscopy results confirmed that AgNPs accumulated heavily in lysosomes and partially colocalized in mitochondria. Additionally, internalization inhibitors were added to the cells, and it was observed that AgNPs could avoid classical drug resistance mechanisms. Furthermore, AgNPs were observed to cause an approximately 60% increase in ROS generation in both cell lines. Cisplatin increased ROS only in A549 cells, while no ROS increase was observed in the A549/DDP. Mitochondrial damage assays showed that AgNPs caused mitochondrial fragmentation and decreased interconnectivity, the level of preservation of the interconnected network structure between mitochondria, in both A549 and A549/DDP cell lines. Additionally, AgNPs reduced basal respiration, maximal respiration, ATP production, proton leak, and non-mitochondrial respiration in both cell lines. In in vivo tests, tumors were induced in mice using the A549 and A549/DDP cell lines and observed for 21 days. Following AgNP treatment, the tumor size decreased from 882 mg in the control group to 292 mg in mice carrying the A549 cell line. Similarly, following AgNP treatment, the tumor size decreased from 805 mg in the control group to 415 mg in mice carrying the A549/DDH cell line. When organ toxicity was measured, no specific toxicity was observed in the heart, liver, kidney, lung, or spleen tissues, and no significant change was observed in the body weight of the mice. Western blot analysis also determined that AgNPs affected pathways such as p53, VEGF, Cyclin 1, and CTR1, and that these effects resulted in the induction of apoptosis and disruption of the cell cycle.

Most of the studies demonstrate that AgNPs exhibit significant dose-dependent anticancer effects in A549 lung cancer cells. These effects include suppression of cell proliferation, induction of apoptosis, inhibition of cell migration, and changes in gene expression levels. These results suggest that AgNPs are promising bionanomaterials for lung cancer treatment.

### 4.3. Colon and Colorectal Cancer

Taati et al. also first performed characterization tests on AgNPs (Ag@Gln-TSC NPs) functionalized with glutamine and thiosemicarbazide conjugation and observed spherical AgNPs [130]. Subsequently, MTT assays were performed to perform anticancer tests of Ag@Gln-TSC NPs. SW480 (human colorectal cancer cell line) and HEK293 cell lines used in these tests were exposed to Ag@Gln-TSC NPs at a concentration of 31.25 µg/mL. A 14.2% decrease in viability was observed for both cell lines. However, at a concentration of 62 µg/mL, the produced AgNPs did not cause significant toxicity with a 9.2% decrease in viability. Ag@Gln-TSC NPs exhibited the highest inhibitory activity at a concentration of 500 µg/mL. At this concentration, the viability of colon cancer cells was reduced by 98.7% and that of normal cells by 83.5%. Subsequent calculations revealed IC_50_ values of 88 µg/mL for SW480 and 186 µg/mL for normal cells. Additionally, ROS level experiments showed that ROS generation was significantly higher in cells treated with Ag@Gln-TSC NPs compared to the control group. Furthermore, the early apoptosis rate in the control group increased from 1.26% to 79.83% with Ag@Gln-TSC NPs application. Moreover, cell cycle analysis revealed a significant arrest at the S phase in cells treated with Ag@Gln-TSC. Caspase-3 activity was also significantly increased by 5.2-fold in SW480 cell lines after Ag@Gln-TSC NPs treatment compared to the control group. Real-time PCR analysis showed that Ag@Gln-TSC NPs increased CASP8 gene expression in SW480 cells, while decreasing the expression of HULC and PPIA4 genes. Suppression of these genes also confirms the anticancer activity of the produced Ag@Gln-TSC NPs. In conclusion, Ag@Gln-TSC NPs demonstrated significant anticancer activity in the SW480 cell line. Furthermore, comprehensive tests (such as cell cycle and gene expression assays) confirmed the anticancer activity of the novel Ag@Gln-TSC NPs.

In the study by Shaaban et al., the anticancer activity of AgNPs synthesized using the *S. enissocaeslis* plant, which are spherical and 32.2 nm in size, was measured [131]. MTT tests were used for this purpose, and the Caco-2 cell line was used as the colon cancer cell line. The viability rates of Caco-2 cells treated with AgNPs at varying concentrations decreased from 99% to 34% after 48 h of exposure. AgNPs showed a very high anticancer potential with an IC_50_ value of 0.156 mg/mL. Additionally, the standard chemotherapy drug cisplatin also showed a cell viability rate of approximately 18% in Caco-2 cells. Following the application, morphological changes such as shrinkage and rounding were observed in the cells. Additionally, the researchers noted that in another study, AgNPs synthesized from another plant treated with AgNPs in the Caco-2 cell line showed a dose-dependent decrease in viability from 98% (12.5 μg/mL) to 4.8% (200 μg/mL).

Bhalla et al. tested the anticancer properties of *E. durans* bacteria, a type of gut microbe, treated with low-concentration AgNPs obtained ready-made using HCT116 in an MTT assay [132]. The supernatant was collected from the AgNP-treated cultures at the 9th hour, and cancer cells were treated with this supernatant. Cell viability decreased by 19% compared to the control group. At the same time, the folate (a multifunctional metabolite) released at this moment corresponds to the time when it is most cytotoxic. Subsequently, the ROS-mediated anticancer effect was tested, and the results showed that while the intracellular superoxide concentration increased by 13% during the mid-log phase of bacterial culture growth, hydroxyl radical levels increased by 48% compared to the control during the late log phase (9th hour). Additionally, it has been noted that increased ROS levels may affect different metabolites and secretion systems. In conclusion, it has been observed that AgNP-treated *E. durans* bacteria can affect cell viability, particularly the viability of colorectal cells, through properties such as inducing ROS production or altering the secretion levels of different metabolites.

Jain et al. evaluated the anticancer activity of AgNPs synthesized from three different types of turmeric (*Curcuma longa*, *Curcuma caesia*, and *Curcuma aromatica*), using the HT-29 (human colon cancer cell line) and the B (SRB) assay for this purpose [133]. The results of the B (SRB) assay showed that all synthesized AgNPs reduced the percentage of viability at increasing concentrations, but this decrease in viability varied depending on the plant from which the AgNP was synthesized. *C. longa* AgNPs were recorded as the most effective anticancer agent, reducing cell viability by 12.6% compared to the control group. AgNPs from other species also exhibited cell viability values ranging from 14.6% to 16.4%. In contrast, the standard drug Adriamycin NPs exhibited the highest viability reduction (1.1%) among all treatment groups. It has also been noted that the active components present in the plant extract may underlie this activity of AgNPs. In summary, the results of the study indicate that AgNPs synthesized from *Curcuma* species may be considered as a potential anticancer agent due to their high antioxidant properties, their ability to cause disruption in cell morphology, the bioactive components in the plant extract, and dose-dependent toxicity.

Birtekocak et al. conjugated AgNPs with TRAIL (TNF-related apoptosis-inducing ligand) due to TRAIL’s anticancer properties [134]. The TRAIL-coated NPs obtained after the synthesis process were named AgCTP NPs. Subsequently, the MTT assay was used to test the anticancer activity of these AgNPs. For this purpose, AgNPs were applied to HT-29 cells at different doses, and AgC (Ag + cysteine) NPs did not cause a significant decrease in cell viability. Similarly, AgCT (Ag + cysteine + TRAIL) and AgCTP (Ag + cysteine + TRAIL + PEG) did not cause a decrease in viability up to doses of 12.5 ng/mL and 6.25 ng/mL, respectively. However, 25 ng/mL AgCTP treatment caused a significant decrease in viability compared to the control group. Subsequently, morphological changes in HT-29 cells were also examined, and it was observed that cells treated with AgCTP exhibited membrane damage, slowed or arrested cell growth, which may be associated with cell cycle arrest and apoptotic symptoms. Additionally, following treatment with the same dose of AgC, AgCT, and AgCTP, AgCT and AgCTP decreased the tendency of HT-29 cells to metastasize. Additionally, the expression levels of proteins such as Bax, Bcl-2, PARP, and clv-PARP in cells treated with AgCTP for 24 h were examined, and differences were observed in the expression levels of Bax, Bcl-2, and clv-PARP.

Concurrently, AgNPs have emerged as promising nanomaterials for the treatment of colon and colorectal cancers due to their potent cytotoxicity and ability to induce apoptosis. Although in vitro findings are promising, further studies are needed to ensure their biosafety and clinical applicability.

### 4.4. Other Cancer Types

Studies on the anticancer activities of AgNPs have generally used colon, colorectal, breast, and lung cancer cell lines. However, research on different cancer types affecting other organs is quite limited. While the biological responses of cancer cells to AgNPs can vary across multiple parameters, measuring their activity against different cancer types will help define AgNPs’ anticancer effects. Additionally, these studies will determine whether AgNPs are a universal anticancer agent or a cancer-type-specific treatment approach.

In a study conducted by Zhang et al., AgNPs were synthesized in situ using tannic acid (TA)-capped gemcitabine (GEM)-loaded poly-L-lactic acid (PLLA) fibers [135]. This system is stated as AAFM (Antibacterial and Anticancer Fibrous Membrane). To evaluate cytotoxicity and anticancer activity of AAFMs, PANC-1 (human pancreatic cancer line) and NIH/3T3 (mouse embryonic fibroblast cell line) have been used in MTT assays. The results of these assays showed that both Ag-containing GEM-PLLA groups and Ag-free GEM-PLLA groups caused high levels of cell death within the first few days. This was explained by the high local drug concentration resulting from rapid drug release from the fibers. Furthermore, the presence of TA-stabilized AgNPs contributed to a synergistic increase in the cytotoxic effect on fibroblasts and pancreatic cancer cells (NIH/3T3 and PANC-1). In long-term analyses, tests performed with leachates obtained from membranes stored in PBS (phosphate-buffered saline) for 30 days showed significantly more cell death and lower cell viability compared to the control group. Additionally, the CCK-8 test also yielded results that support these findings. When all findings were combined, it was concluded that the developed multifunctional membrane has significant potential in preventing tumor recurrence by exhibiting short-term anticancer effects.

Asif et al. conducted a comprehensive study using different kinds of cancer lines, such as A2780, A2780cis, SK-OV-3 (ovarian cancer cell lines); U-87 MG (glioblastoma cell line), and MRC-5 (human fibroblast cell line) [136]. Cytotoxicity and anticancer activity assays showed that AgNPs exhibit high cytotoxicity value (IC_50_ = 0.0003–0.28 µg/mL) against A2780, A2780cis, SK-OV-3, U-87 MG, and MRC-5 cell lines after 96 h of treatment. Additionally, it has been noted that AgNPs exhibit higher anticancer activity than cisplatin, although at varying rates. Furthermore, the delayed apoptosis rate was determined to be 79%, which was confirmed by cytochrome c release associated with mitochondrial damage. Following exposure of cancer cells to AgNP, an eight-fold increase in the HO-1 gene and suppression of Akt phosphorylation led to cell death via oxidative stress. Furthermore, high efficacy was observed at concentrations of 1.1–7 µg/mL in HGSOC organoids derived from patient samples, while no toxic effects were observed in normal liver organoids. Following exposure of cancer cells to AgNP, an eight-fold increase in the HO-1 gene and suppression of Akt phosphorylation led to cell death via oxidative stress. Furthermore, high efficacy was observed at concentrations of 1.1–7 µg/mL in HGSOC organoids derived from patient samples, while no toxic effects were observed in normal liver organoids. AgNPs have demonstrated potent anticancer effects, particularly against ovarian cancer and cisplatin-resistant cell lines, while remaining biocompatible with normal organoids. These results suggest that this nanomaterial could be used as an alternative to cisplatin-based therapies, acting as a selective, oxidative stress-based anticancer agent.

Guo et al. tested the anticancer activity of AgNPs synthesized from *Syzygium aromaticum*, which are 50 nm in size and spherical in shape, against 32D-FLT3-ITD (human leukemia cell line) [137]. In the MTT assays conducted for this purpose, AgNPs were applied at doses of 1–1000 µg/mL, and the resulting IC_50_ value was determined to be 162 µg/mL. Additionally, tests following treatment observed a significant increase in LDH release, indicating significant membrane damage. Similarly, ROS levels were measured after treatment, and a significant increase was observed in the AgNP-treated leukemia cells, while no significant change was reported in the PCS-800-011 (primary peripheral blood mononuclear cells) group. Moreover, flow cytometry revealed that the percentage of apoptosis increased significantly in 32D-FLT3-ITD cells treated with AgNP, but no significant change was observed in PCS-800-011 cells. Additionally, increased expression of Bax and caspase-3 genes was observed in AgNP-treated 32D-FLT3-ITD cells, while the Bcl-2 gene and PI3K, AKT, and mTOR pathways were suppressed. These effects facilitate cell apoptosis and inhibit survival systems. Taking a broader view, it has been shown that AgNP could be both a selective and potent anticancer agent against leukemia, one of the cancer types that deserves more attention in the literature, alongside AgNP.

In another study conducted by Dervişoğlu et al., PrAgNPs produced using Bingöl propolis were first subjected to characterization tests [138]. As a result of these tests, their size and shape were found to be 14.45 ± 3.44 nm and spherical, respectively. WST-1 assay was used to test the anticancer activity of these PrAgNPs and EEPs (ethanol extract of propolis) against PC-3 (human prostate cancer cell line). PrAgNPs showed a high dose-dependent cytotoxicity with an IC_50_ value of 24.2 ± 0.8 µg/mL, while EEPs did not show significant effects at low doses. However, it has been shown to cause significant cytotoxicity at 125 µg/mL, and it has been suggested that this is due to EEP being able to pass through the cell membrane more easily when used in combination with AgNPs. Additionally, Western blot results have demonstrated that PrAgNP application activates the mitochondrial apoptosis pathway by increasing cytochrome c and procaspase-3 activity.

In a study conducted by Thakkellapati et al., AgNPs synthesized using ammonium hydroxide, described as spherical and 200–1000 nm in size, were tested for their photothermal patch function and anticancer activity [139]. The patches, produced with a diameter of 100 mm and a thickness of 1.5 mm, consist of a PVAc layer, a silver polymer composite, and a second PVAc layer. It was reported that the produced patch could reach 62 degrees within 5 min under infrared light (IR). MTT assays were used to measure the anticancer efficacy of the produced patch against U251 (human glioblastoma multiforme cell line) cells. In the control groups (light-only group and patch-only group), cell viability was not significantly reduced. However, when the photothermal effect of the patch was tested, the patch and IR light combination reduced cell viability to 19%, indicating strong photothermal cytotoxicity. Additionally, examination of cell morphology revealed membrane disruption. Furthermore, the DCFH-DA assay confirmed that the patch induces photothermal ROS production. In summary, the produced patch has potential in the treatment of brain cancer using heat due to its local heat generation and ROS production caused by the photothermal effect, as well as its reusability and ease of application.

Moshrefi and Hosseini, who suggested that AgNPs could be a potential treatment for gastric cancer, evaluated AgNPs with 99% purity and 50 nm size in both in vivo and in vitro conditions [140]. First, in vitro tests were conducted using MKN45 cells, a human gastric adenocarcinoma cell line, and then MTT assays were performed. AgNPs at different concentrations were incubated for 48 h in the MTT assays, and the resulting IC_50_ value was measured as 22.4 µg/mL, indicating significant and potent anticancer activity. In the in vivo tests, 20 eight-week-old female mice were divided into four different groups. Two of these groups received 75 mg/kg AgNP and 150 mg/kg AgNP, while the other two were control and sham groups. The tumors created by the MKN45 cells injected into the hind legs of these mice were observed throughout the 4-week treatment period. According to the results, AgNPs at a concentration of 150 mg/kg accumulated in the tumor tissue and significantly reduced tumor size. Additionally, while cell proliferation was evident in the control and sham groups, cell proliferation decreased, and necrosis rates increased in the AgNP-treated groups. Additionally, increasing AgNP concentration increased the expression of the BAX and BCL2 genes and decreased CXCR1 gene expression. Taken together, researchers stated that AgNPs have a high potential for treating gastric cancer, with strong anticancer activity through apoptosis-inducing mechanisms.

In another study to test the anticancer activity of AgNPs, chemically synthesized AgNPs loaded with GEM were evaluated in vivo to determine whether GEM-AgNPs could serve as a nanotechnological drug delivery system [141]. For this purpose, the toxic side effects of GEM-AgNPs were tested on 40 male rats. The rats were randomly assigned to four groups: GEM, AgNPs, GEM-AgNPs, and the control group. The results showed that all of the control group survived, while the survival rate in the GEM group was 8/10. Additionally, weakness, decreased mobility, weight loss, and hair loss were observed in the GEM-AgNP group. While AgNPs alone did not cause significant toxicity, hematological, biochemical, and tissue damage were significantly reduced in the GEM-AgNPs group compared to GEM. In vitro, MTT assays were performed using the HepG2 cell line. GEM, AgNPs, and GEM-AgNPs were applied to the cells and incubated. The results showed IC_50_ values of 24.19 µg/mL, 50.6 µg/mL, and 13.63 µg/mL, respectively, indicating the strongest cytotoxicity in the GEM-AgNP group. Furthermore, apoptosis and necrosis tests revealed that GEM-AgNPs induced the most apoptotic cell death. Cell cycle analyses also support these findings. In conclusion, after controlling the toxic side effects of GEM-AgNPs in vivo and demonstrating their anticancer activity, the potential for use in drug delivery systems and as anticancer agents has been extensively investigated.

**Figure 3:**
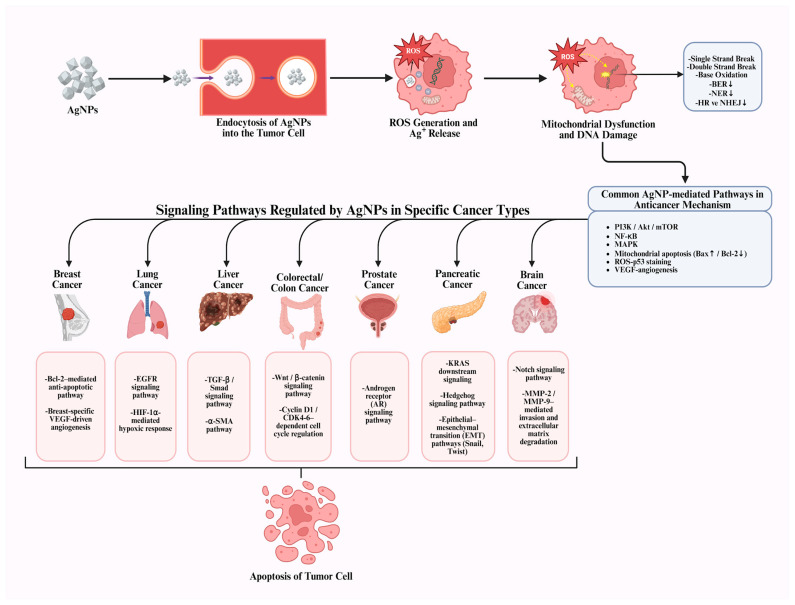
Cancer type-specific mechanisms underlying AgNP-mediated anticancer activity [8].

In summary, recent in vivo and in vitro studies indicate that AgNPs hold promise as selective and multifunctional anticancer agents against various cancer types, including breast, lung, liver, colorectal, and prostate cancer, offering new therapeutic opportunities beyond conventional treatments (Figure 3). It is widely accepted that AgNPs exhibit cytotoxicity, a characteristic consistently demonstrated across numerous experimental studies. Current evidence indicates that, through appropriate surface modifications, functionalization strategies, and controlled synthesis approaches, this toxicity can be selectively redirected toward cancer cells. Nevertheless, critical challenges persist, including nonspecific cytotoxicity, insufficient tumor selectivity, and long-term in vivo safety concerns. To overcome these limitations and safety concerns, further studies should focus on dose optimization, standardized NP design, targeted delivery systems, and comprehensive in vivo toxicity and pharmacokinetic evaluations. Overall, recent research trends indicate a focus on engineering safer and more selective AgNP-based platforms, suggesting that AgNP modification may play a key role in translating their anticancer potential into clinically viable therapies.

## 5. Challenges, Limitations, and Concerns About AgNPs as Anticancer Agents

AgNPs owe their anticancer activity to their unique physicochemical properties [142,143]. Therefore, even small changes in AgNP parameters such as size, shape, and surface chemistry can significantly affect the cellular uptake rate, ROS production, and the effectiveness of different cytotoxic mechanisms [144,145].

The primary anticancer activity of AgNPs is related to their induction of ROS production, negative effects on the cell cycle, mitochondrial dysfunction mechanisms, and membrane damage. Moreover, AgNPs are known to affect cytokine secretion, which can modulate immune responses and inflammatory signaling, enhancing cytotoxic outcomes [146]. While these mechanisms highlight the anticancer properties of AgNPs, the same effects can also lead to organ damage and other toxic effects when applied to healthy cells. These phenomena raise questions about the use of AgNPs as anticancer agents [44].

For example, a change towards cancer formation in lung cells exposed to low doses of AgNP for long periods was observed in an RNA-sequencing study by Gliga et al. [147]. In the immunotoxicity study conducted by Bi et al., different parameters were evaluated, and it was observed that metal NPs can cause oxidative stress and inflammation. In long-term tests, it was shown that they can reduce the number of NK cells, and specifically AgNPs trigger responses such as ATF-6 degradation, increased ER stress, and NLRP-3 inflammasome activation. In addition, particles with a size of 20 nm can accumulate heavily in the liver, kidney, and spleen [148]. According to another study, in vivo research conducted on Swiss mice showed that AgNPs encapsulated in montmorillonite did not cause systemic toxicity at the tested doses, but they remained in the bloodstream for a long time and crossed the blood–brain barrier, affecting neurotransmission in healthy brain tissue. This finding demonstrates that although AgNPs do not exhibit the classic risk of toxicity, they can cause functional impairments in healthy tissues of vital organs such as the brain [149]. Another study observed that AgNPs inhaled during pregnancy crossed the placental barrier in mice and reached placental and fetal tissues. This exposure was found to increase inflammatory cytokine expression in healthy tissues and cause a decrease in estrogen levels. The findings demonstrate that AgNPs can affect not only healthy adult tissues but also highly sensitive tissues such as the placenta and developing fetus [150]. Furthermore, Hadrup et al. have mentioned that orally ingested AgNPs are absorbed to a certain extent in mammals and accumulate at high levels, particularly in the stomach and intestines. In animal studies, AgNP exposure is known to lead to multiple systemic toxic effects in healthy tissues, including weight loss, behavioral changes, changes in liver enzymes, cardiac enlargement, and immunological effects [151]. Toxic activities, which can also cause cytotoxicity, have been reported to lead to organ dysfunctions and toxicity, as mentioned, and these activities narrow the therapeutic window of AgNPs in anticancer applications. Despite their potent anticancer profile, the side effect mechanisms of AgNPs raise concerns about the therapeutic potential of their potent biological activities [146].

However, this toxicity can be reduced through various modifications, which is one of the greatest advantages of using AgNPs. A study has shown that the toxic effects of AgNPs can be significantly altered by surface functionalization. Unfunctionalized AgNPs increase the release of Ag^+^ ions within the cell, leading to the production of excess ROS and consequent oxidative stress and apoptosis. In contrast, coating AgNPs with PEG or BSA limited Ag^+^ release, reduced ROS formation, and preserved cellular antioxidant defenses. Thus, the activation of mitochondrial apoptotic pathways was suppressed, and cell damage was significantly reduced [152]. Another study comparatively examines the toxic effects of uncoated and surface-coated AgNPs. The findings revealed that uncoated AgNPs significantly reduced cell viability even at low concentrations, with smaller particles exhibiting higher cytotoxicity. In contrast, AgNPs coated with citrate and PVP showed toxic effects only at higher concentrations. Surface coating limited the capacity of AgNPs to generate oxidative stress, largely preserving GSH and SOD levels. However, PVP-coated AgNPs were observed to generate a greater increase in cytokines compared to citrate-coated ones [153]. In conclusion, besides optimizing the size and shapes of AgNPs, surface functionalization emerges as a critical strategy for mitigating the toxic effects of AgNPs, but due to the nature of AgNPs, the selection of appropriate coating agents and rigorous toxicity checks before proceeding to clinical trials are necessary.

While the anticancer potential of AgNPs has attracted considerable attention, transferring these systems from the laboratory to the industrial scale presents significant challenges for different types of industries. In particular, the difficulty of standardizing plant-based and green synthesis methods is emerging as a limiting factor in the AgNP production process [154]. It is quite difficult to maintain the properties of AgNPs obtained under controlled laboratory conditions when transferred to large-scale production. In addition, many uncertainties can arise throughout the process. In particular, the inconsistency of various parameters that play a role in anticancer activity, such as size, shape, surface chemistry, and stability, as production volumes increase, makes standardization difficult [155]. These standardization challenges complicate the physicochemical properties affected by different synthesis methods or the production of stable NPs. This can lead to the production of NPs exhibiting unexpected levels of biological activity.

A study conducted by Gomes et al. provides a comprehensive assessment confirming these concerns. Testing AgNPs obtained from different synthesis protocols in A549 lung cancer cells, the researchers observed early apoptotic cell death at high doses, metabolic disruption due to oxidative stress, and time- and dose-dependent cytotoxicity. These biological effects reported in the study highlight the need to carefully manage potential toxicity risks, as well as the anticancer activity of AgNPs, and support safety concerns in the literature [156].

The biodistribution of AgNPs is also a difficult-to-control, multivariate, and unpredictable process. This leads to uncertainty regarding the pharmacokinetics of AgNPs. As previously mentioned, AgNPs can accumulate in various tissues and organs, making accumulation in target tumor tissues more difficult and increasing the risk of long-term persistence in the reticuloendothelial system (RES) organs [157]. Therefore, rapid clearance from circulation, strong retention in RES organs, and low accumulation in target tissues are the main pharmacokinetic challenges that limit the clinical efficacy of AgNPs [158].

Finally, one of the biggest obstacles to transitioning AgNPs to clinical applications for anticancer purposes is the lack of a clear and standardized regulatory framework specifically for AgNPs. Furthermore, both the FDA (Food and Drug Administration) and the ECHA (European Chemicals Agency) have general regulatory guidelines for nanomaterials. For example, the FDA, in its “Drug Products, Including Biological Products, that Contain Nanomaterials” guideline, clearly states that drugs containing NPs require more rigorous examination than standard drugs regarding manufacturing, quality, toxicity, biodistribution, and characterization [159]. It also states that more data and testing are required compared to other standard drugs, and that more PK/PD (pharmacokinetic-pharmacodynamic), ADME (Absorption, Distribution, Metabolism, and Excretion), and toxicity studies are required compared to standard drugs. In the ECHA, however, limitations specific to the biomedical applications of AgNPs also exist [160]. Although REACH (Registration, Evaluation, Authorization and Restriction of Chemicals) includes nanomaterials within the definition of “substance,” it is not clearly defined how critical parameters such as NPs size, aggregation tendency, and surface properties will be addressed during the registration and evaluation process [161]. Additionally, insufficient data are available regarding the target tissue specificity, toxicity-benefit balance, and long-term biocompatibility of AgNPs to enable clinical application. These deficiencies in the EU regulatory system could lead to delays in the authorization process.

## 6. Conclusions

AgNPs, owing to their physicochemical and biological properties, can offer a strong anticancer effect, making them an alternative treatment option to conventional cancer treatment methods. Conventional cancer treatments, such as chemotherapy and radiotherapy, often cause serious toxic effects in healthy cells due to their low selectivity, increasing treatment-related side effects such as susceptibility to infections, neuropathy, and leukopenia [162,163]. However, AgNPs, on the other hand, can provide efficacy even at lower doses due to their potential to exhibit more selective effects against tumor cells and their ability to activate multiple cellular mechanisms simultaneously, including oxidative stress induction, mitochondria dysfunction, and induction of apoptosis via DNA damage and cell cycle arrest. Furthermore, AgNP-based approaches can be considered a promising alternative to conventional treatments in terms of reducing the development of drug resistance. This advantage is based on the ability of AgNPs to target multiple cellular signaling pathways, making it more difficult for cancer cells to develop resistance. In contrast, chemotherapeutic agents can lead to the development of resistance over time by focusing on a specific molecular target, limiting long-term treatment effectiveness. However, their cytotoxicity has led to debates about the use of this potent anticancer agent in clinical trials. While many studies have demonstrated selective cytotoxicity of AgNPs, especially compared to some chemotherapeutic drugs, some studies have observed that AgNPs are also toxic to healthy cells and reduce their viability. To overcome this, researchers have shown that selective cytotoxicity can be increased through methods such as using green synthesis methods, modifying AgNPs, using them in combination with different drugs, or surface functionalization. AgNPs with proven cytotoxic properties have been tested on different cancer cell lines in studies and have been observed to exhibit anticancer properties against various cancer cell lines from many different organs. Studies on breast, lung, colon, and prostate cancer cell lines constitute the majority of all studies examining the AgNP-anticancer relationship. In addition, studies on cancer types such as brain, liver, skin, and leukemia are available in the literature. However, it is also true that preclinical studies are needed to prove that AgNPs can truly offer an alternative cancer treatment and to enable their use in clinical trials. This includes testing them on different cancer types, increasing and diversifying in vivo studies, and mass production under optimal conditions with minimal toxicity. In this context, the safe use of AgNPs is closely related to regulatory frameworks established by the FDA and ECHA, which are based on physicochemical characterization and toxicity analyses, as well as regulations such as REACH. These regulatory approaches are crucial for clearly establishing dose optimization, exposure level, and long-term safety at the preclinical stage. However, fitting with the European Medicines Agency (EMA) requirements, which include the entire medicinal product development process and are based on clinical safety, efficacy, and quality standards, is inevitable for AgNP-based approaches to be transferred to clinical applications. Overall, this multi-stage regulatory process paves the way for AgNPs to be considered a safe and sustainable therapeutic option in cancer treatment.

In conclusion, the tumor-killing effects of AgNPs have been demonstrated by numerous studies in the literature, but their cytotoxic properties continue to raise safety concerns. With proper synthesis and modification methods and strict safety measures, AgNPs exhibit high anticancer efficacy and have the potential to be a powerful anticancer treatment. However, increasing the number and variety of studies in the literature is crucial to demonstrate whether AgNPs can be used not only in laboratory settings but also in actual clinical trials.

## Figures and Tables

**Table 2:** Recent anticancer studies of AgNPs on various types of cancers.

Cancer Type	AgNP Property	Main Results	Reference
Breast Cancer	- Green synthesized AgNPs from *Fusarium equiseti* extract. - Average size of 50. - Spherical in shape - Fungal-metabolite capped.	- Dose-dependent cytotoxicity against MCF-7 cells. - IC_50(MCF-7)_ = 24.38 µg/mL. - Decreased cell viability. - ROS-related apoptotic effects suggested.	[103]
Breast Cancer	- Chemically synthesized AgNPs. - Average size of 10.2 ± 2.5 nm. - Quasii-spherical in shape. - Citrate-coated.	- AgNPs agglomerated in the cytoplasm of 4T1 cells. - IC_50(4T1)_ = 74.4 μM. - AgNPs decreased the expression of Ccl2, Ccl22, Cd206, Tgf-β, Nos2, Mmp9 genes. - AgNP-treated 4T1 cells suppressed macrophage migration.	[104]
Breast Cancer Prostate Cancer	- Green synthesized AgNPs from *Endophytic Streptomyces* sp. KE4D ve *Bacillus safensis* KE4K. - 5–55 nm in size. - KE4D: spherical, KE4K: various shapes. - Cyclopropane, acetic acids, and fatty acids coated.	- IC_50(MCF-7)_ < 3.5 µg/mL - IC_50(DU-145)_ < 2.5 µg/mL - KE4D-derived AgNPs synthesized in medium 5294 showed the highest QSI activity with 85.12%. - KE4K-derived AgNPs showed superior antimicrobial activity, particularly against Gram-negative bacteria.	[94]
Breast Cancer	- Commercial AgNP formulation. - Average size of 68 nm.	- AgNP and tamoxifen showed synergistic effects. - AgNP + tamoxifen combination produced antiproliferative and cytotoxic effects in both cell lines. - The combination induced a 96% increase in ROS in MDA-MB-231 cells and a 52% increase in MCF-7 cells. - The level of genotoxicity remained low. - Increased expression of pro-apoptotic and oxidative stress-inducing genes.	[96]
Breast Cancer	- Green-synthesized AgNPs were produced using *Microalga Desmochloris edaphica* through both biomass extract (DBio) and cell-free supernatant (DSup). - Size ranging from 12 to 15 nm. - Quasi-spherical in shape. - Algal proteins, fatty acids, and polysaccharides were capping agents.	- Both AgNP types showed strong cytotoxic activity against MCF-7 breast cancer cells. - Low toxicity toward normal Vero kidney cells, indicating good biocompatibility. - Potent inhibition against *Staphylococcus aureus*, *Bacillus subtilis*, and *Shigella flexneri*.	[105]
Lung Cancer	- AgNPs synthesized from kenaf seed (KS) extract. - Size ranging from 7 to 11 nm. - Spherical in shape. - 19.6 µg/L Ag^+^ release from KS@AgNPs. - KS coated.	- KS@AgNPs showed a strong antiproliferative effect against A549 cells. - High doses were non-toxic to normal NIH3T3 cells, indicating good selectivity. - Combination with ampicillin produced a synergistic antimicrobial effect.	[106]
Lung Cancer	- Green synthesized AgNPs produced using *Momordica charantia* (bitter melon) fruit extract. - Size ranging from 1 to 13.85 nm. - Spherical in shape.	- AgNPs showed strıng and dose-dependent cytotoxicity against A549 cells. - IC_50(A549)_ = 51.93 µg/mL - IC_50(HOP-62)_ = 76.92 µg/mL - *M. charantia*-capped AgNPs were observed to be more biocompatible than the raw fruit extract itself.	[107]
Colorectal Cancer Lung Cancer	- Green synthesized Sg-AgNPs produced using Siberian ginseng (*Eleutherococcus senticasus*) extract.	- Sg-AgNPs significantly reduced the viability of HT-29 and A549 cells. - Cytotoxic effect stronger than commercial AgNPs and cisplatin. - Morphological nuclear changes induced at 10 µg/mL. - Higher ROS levels in both cell lines than in commercial AgNPs and cisplatin. - Activation of the p53 MAPK pathway was confirmed - In HT-29, the CASP3 gene and Caspase-3 protein were activated. - In A549, only Caspase-3 protein activation was observed. - Overall findings indicate apoptosis triggered through the ROS-mediated Caspase-3/p38 MAPK axis.	[108]
Liver Cancer Lung Cancer	- Green synthesized AgNPs produced from *Fucoidan* extracted from *Fucus vesiculosus*. - Size ranging from 4 to 45 nm. - Generally spherical in shape.	- As AgNP concentration increased, HepG2 and A549 cell viability gradually decreased, and cytotoxicity became evident at high concentrations. - Significant antioxidant activity (~84% DPPH scavenging) at 10 µg/mL AgNP concentration.	[109]
Colorectal Cancer	- Green synthesized MC-AgNPs produced from *Myrtus communis* (MC). - Biphasic NPs: bio-molecule layer + metallic Ag^0^ layer.	- Exhibited remarkable cytotoxicity against HCT-116 cells. - IC_50_ = 5.99 µg/mL (24 h). - The therapeutic effect of MC-AgNPs was two-fold higher than that of doxorubicin.	[110]
Colorectal Cancer	- Green synthesized AgNPs produced from *Anthemis atropatana* extract. - Average size of 38.89 nm. - Spherical in shape.	- AgNPs demonstrated dose-dependent cytotoxicity against HT-29 cells. - IC_50_ = 4.88 μg/mL. - Maximum cytotoxic effect at 100 µg/mL, significantly different from the control group. - Induced apoptosis.	[111]
Liver Cancer	- Green synthesized AgNPs produced from *Salmonella entrica* supernatant. - Size ranging from 7.18 to 13.24 nm. - Spherical in shape.	- AgNPs demonstrated dose-dependent cytotoxicity against Hep-2 cells. - IC_50_ = 80 µg/mL - Strong antioxidant activity in the DPPH test range of 10–40 µg/mL. - AgNPs also showed antibacterial and antidiabetic activity.	[112]
Pancreatic Ductal Adenocarcinoma(PDAC)	- AgNPs are synthesized using electrochemical methods. - Average size of 9.04 nm. - Spherical in shape.	- AgNPs nearly destroyed viability in BxPc-3, PANC-1, and MIA-PaCa2 cell lines. - Non-malignant cells (CRL-4023 and LX-2) remained largely resistant. - α-lipoic acid administration protected normal cells from AgNP toxicity but not PDAC cells. - AgNPs reduced tumor growth in chicken eggs (xenograft model). - α-lipoic acid prevented the liver toxicity of AgNPs in chicken embryos.	[113]
Lung Cancer Pancreatic Cancer Prostate Cancer	- Green synthesized AgNPs produced from *Anemone coronaria* bulb extract. - Average size of 29 nm. - Spherical in shape.	- Significant cytotoxicity towards cancer cells, while sparing normal cells. - IC_50(A549)_ = 9.15 µg/mL, IC_50(Mia-PaCa2)_ = 9.87 µg/mL, IC_50(PC-3)_ = 12.1 µg/mL. - Apoptosis confirmed by Bax upregulation and Bl-2 downregulation. - Cell cycle arrest observed. - Gene expression analyses support apoptotic pathway activation. - AgNPs also showed strong antibacterial activity.	[114]
Acute T-cell Lymphoblastic Leukemia	- Green synthesized AgNPs produced from *Vicia faba* seed coat aqueous extract. - Average hydrodynamic diameter of 23.14 ± 0.20 nm. - Spherical morphology.	- Strong dose-dependent cytotoxicity against leukemia cells. - IC_50_ = 2.27 mg/mL. - A high percentage of cells in sub-G1 confirms apoptotic cell death. - Flow cytometry using the Annexin V assay confirmed that increasing AgNP concentration increased apoptosis induction.	[115]
Ovarian Cancer	- Three types of green-synthesized AgNPs were produced from *Asplenium dalhousiae* leaf aqueous, chloroform, and n-hexane extract. - Size ranging from 34.43 to 59.43 nm. - Spherical in shape.	- Potent cytotoxicity against A2780 ovarian cancer cells. - Aqueous AgNPs IC_50_: 15.76 µg/mL - n-hexane AgNPs IC_50_: 9.11 µg/mL - n-hexane extract-AgNPs also showed the strongest antibacterial effect.	[116]
Lung Cancer Colon Cancer Breast Cancer Prostate Cancer	- Green synthesized AgNPs produced from *Anchusa officinalis* L. seed coat aqueous extract. - Average size of 28.5 nm. - Spherical in shape.	- Dose-dependent inhibition on LnCap, Caco-2, MDA-MB-231, and A549 cell lines. - Significant suppression, especially at 25 µg/mL AgNP concentration. - The lowest EC_50_ value is 15.15 µg/mL for A549 cells. - As concentration increased, inhibition of healthy HEK-293 cells decreased, indicating relative safety and selective toxicity toward cancer cells.	[117]
Skin Cancer	- Green synthesized AgNPs produced from *Cajanus trinervius* leaf extract. - Average size of 18.20 nm. - Spherical in shape.	- Dose-dependent cytotoxicity observed. - Both CT leaf extract and CT-AgNPs decreased cell viability, but CT-AgNPs showed a stronger cytotoxic effect. - IC_50(A431)_ = 45.53 μg/mL - AO/EB dual staining confirmed that apoptosis is the main pathway of cell death. - Significant intracellular ROS elevation after 24 h CT-AgNP treatment.	[118]
Liver Cancer	- Green synthesized from the *Lactobacillus acidophilus* strain RBIM. - Size ranging from 17 to 19 nm. - Spherical in shape. - Surface functional groups: Silanols, Carboxylates, Phosphonates, Siloxanes.	- Strong anticancer activity against HepG2 cells. - IC_50(HepG2)_ = 4.217 µg/mL - IC_50(WI-38)_ = 154.1 µg/mL - AgNPs showed high selectivity. - Upregulation of genes such as TNF-α, IL-33, and AMPK and an increase in glutathione levels were detected. - Decreased expression was observed in mTOR, BCL-2, MMP-9, and α-SMA genes. - The treatment suppressed cell proliferation and modulated the autophagy process.	[97]
Skin Melanoma Cancer	- Green synthesized AgNPs produced from *Laurus nobilis* leaf extract. - Size ranging from 28 to 42 nm. - Spherical in shape.	- Green synthesized AgNPs increased the viability of A375 cells, but at the same AgNP concentrations, HaCaT cells were almost not damaged. - Colloidal AgNPs were more toxic but not selective. - Mechanisms such as DNA damage, oxidative stress, ER stress, mitochondrial degradation, apoptosis, and autophagy activation induce death. - Green AgNPs potently suppress oncogenes such as SP1, PTBP3, and DSG2.	[119]

## Data Availability

No new data were created or analyzed in this study.
